# d-Serine Degradation by Proteus mirabilis Contributes to Fitness during Single-Species and Polymicrobial Catheter-Associated Urinary Tract Infection

**DOI:** 10.1128/mSphere.00020-19

**Published:** 2019-02-27

**Authors:** Aimee L. Brauer, Ashley N. White, Brian S. Learman, Alexandra O. Johnson, Chelsie E. Armbruster

**Affiliations:** a Department of Microbiology and Immunology, Jacobs School of Medicine and Biomedical Sciences, State University of New York at Buffalo, Buffalo, New York, USA; University of Kentucky

**Keywords:** d-amino acid, *Enterococcus faecalis*, *Escherichia coli*, *Morganella morganii*, *Proteus mirabilis*, *Providencia stuartii*, bacteremia, catheter, polymicrobial, serine, urinary tract, urine

## Abstract

Proteus mirabilis is a common cause of catheter-associated urinary tract infection (CAUTI) and secondary bacteremia, which are frequently polymicrobial. We previously utilized transposon insertion-site sequencing (Tn-Seq) to identify novel fitness factors for colonization of the catheterized urinary tract during single-species and polymicrobial infection, revealing numerous metabolic pathways that may contribute to P. mirabilis fitness regardless of the presence of other cocolonizing organisms. One such “core” fitness factor was d-serine utilization. In this study, we generated isogenic mutants in d-serine dehydratase (*dsdA*), d-serine permease (*dsdX*), and the divergently transcribed activator of the operon (*dsdC*) to characterize d-serine utilization in P. mirabilis and explore the contribution of this pathway to fitness during single-species and polymicrobial infection. P. mirabilis was capable of utilizing either d- or l-serine as a sole carbon or nitrogen source, and *dsdA*, *dsdX*, and *dsdC* were each specifically required for d-serine degradation. This capability was highly conserved among P. mirabilis isolates, although not universal among uropathogens: Escherichia coli and Morganella morganii utilized d-serine, while Providencia stuartii and Enterococcus faecalis did not. d-Serine utilization did not contribute to P. mirabilis growth in urine *ex vivo* during a 6-h time course but significantly contributed to fitness during single-species and polymicrobial CAUTI during a 96-h time course, regardless of d-serine utilization by the coinfecting isolate. d-Serine utilization also contributed to secondary bacteremia during CAUTI as well as survival in a direct bacteremia model. Thus, we propose d-serine utilization as a core fitness factor in P. mirabilis and a possible target for disruption of infection.

**IMPORTANCE** Urinary tract infections are among the most common health care-associated infections worldwide, the majority of which involve a urinary catheter (CAUTI). Our recent investigation of CAUTIs in nursing home residents identified Proteus mirabilis, *Enterococcus* species, and Escherichia coli as the three most common organisms. These infections are also often polymicrobial, and we identified Morganella morganii, *Enterococcus* species, and Providencia stuartii as being more prevalent during polymicrobial CAUTI than single-species infection. Our research therefore focuses on identifying “core” fitness factors that are highly conserved in P. mirabilis and that contribute to infection regardless of the presence of these other organisms. In this study, we determined that the ability to degrade d-serine, the most abundant d-amino acid in urine and serum, strongly contributes to P. mirabilis fitness within the urinary tract, even when competing for nutrients with another organism. d-Serine uptake and degradation therefore represent potential targets for disruption of P. mirabilis infections.

## Abstract (precis)

Urinary tract infections are among the most common health care-associated infections worldwide, the majority of which involve a urinary catheter (CAUTI). Our recent investigation of CAUTIs in nursing home residents identified Proteus mirabilis, *Enterococcus* species, and Escherichia coli as the three most common organisms. These infections are also often polymicrobial, and we identified Morganella morganii, *Enterococcus* species, and Providencia stuartii as being more prevalent during polymicrobial CAUTI than single-species infection. Our research therefore focuses on identifying “core” fitness factors that are highly conserved in P. mirabilis and that contribute to infection regardless of the presence of these other organisms. In this study, we determined that the ability to degrade d-serine, the most abundant d-amino acid in urine and serum, strongly contributes to P. mirabilis fitness within the urinary tract, even when competing for nutrients with another organism. d-Serine uptake and degradation therefore represent potential targets for disruption of P. mirabilis infections.

## INTRODUCTION

Proteus mirabilis is a motile Gram-negative bacterium most commonly known for its unique bull’s-eye swarming pattern, its potent urease enzyme, and for causing catheter-associated urinary tract infection (CAUTI) (1). Indeed, P. mirabilis is one of the most common causes of CAUTI, particularly in long-term-care facilities (2–5). CAUTIs caused by P. mirabilis can result in the formation of bladder and kidney stones (urolithiasis), permanent renal damage and scaring, dissemination to the bloodstream, and even death (6–10). P. mirabilis CAUTIs are also frequently polymicrobial (2, 5, 11, 12), which has been shown experimentally to increase the risk of urolithiasis and secondary bacteremia (13, 14).

Over the past 3 decades, there have been numerous investigations into the factors harbored by P. mirabilis that contribute to fitness and virulence within the urinary tract (see reference 1 for review). As part of this endeavor, there have been several genome-wide studies for the identification of novel fitness factors, including three signature-tagged mutagenesis studies, an *in vivo* transcriptome study in the murine model of ascending urinary tract infection (UTI) (15–18), and one saturating transposon insertion-site sequencing (Tn-Seq) study in a murine model of CAUTI (19). An important outcome of these studies has been the identification of numerous metabolic pathways that appear to provide niche-specific fitness within the urinary tract, many of which may represent potential therapeutic targets that would not have otherwise been suspected to play a role during infection.

One metabolic pathway identified in these genome-wide screens is the d-serine degradation pathway, encoded by *dsdXA* and *dsdC*. d-Serine dehydratase (DsdA, also known as d-serine ammonia-lyase) catalyzes the deamination of d-serine to pyruvate and ammonium, DsdX is a d-serine permease that facilitates d-serine import, and DsdC is a LysR family transcriptional regulator that is divergently transcribed from the d-serine degradation operon. As deamination of d-serine produces pyruvate and ammonium, bacteria harboring *dsdA* are often capable of utilizing d-serine as a sole source of carbon and nitrogen (20–22). High concentrations of d-serine can also be bacteriostatic due to perturbation of the biosynthetic pathways for l-serine and pantothenate (vitamin B_5_) (23). Thus, d-serine degradation has the potential to increase bacterial fitness in two ways: (i) providing an additional source of carbon and nitrogen and (ii) detoxification of other important pathways.

In humans and mice, d-serine is the most prevalent d-amino acid in urine (0.03 to 1 mM) and serum (0.02 to 3 µM) (24–27), and the ratio of d-serine to l-serine is approximately 1:1 in urine and 1:100 in serum (26, 27). Due to the high concentration of this d-amino acid in urine, degradation has been hypothesized to provide a significant advantage to organisms that colonize the urinary tract. For instance, in Escherichia coli, the genes for d-serine degradation are most prevalent in uropathogenic isolates (22, 23). Similarly, the ability to degrade d-serine also distinguishes Staphylococcus saprophyticus from other *Staphylococcus* species that are less likely to cause UTI (such as S. epidermidis and S. aureus) (21, 28).

In P. mirabilis*, dsdA* is upregulated during ascending UTI (18), and *dsdA* and *dsdC* were identified as candidate fitness factors for bladder and kidney colonization during both single-species and polymicrobial CAUTIs (19). The generation of pyruvate and ammonia through d-serine degradation can perturb central metabolism in other bacterial species, particularly in the presence of a preferred carbon source (such as glucose) (21, 23). However, data from genome-wide screens indicate that P. mirabilis favors pyruvate catabolism within the urinary tract via pyruvate dehydrogenase and dihydrolipoamide acetyltransferase (encoded by *poxB* and *aceEF*) (16, 18, 19). Based on these data, we hypothesized that d-serine degradation would offer P. mirabilis a fitness advantage within the urinary tract by providing an additional carbon and nitrogen source and that this pathway may further provide a niche-specific advantage that allows P. mirabilis to compete better with other colonizing organisms during polymicrobial infection. Furthermore, we proposed that d-serine utilization contributes to the ability of P. mirabilis to disseminate and survive within the bloodstream. In testing this hypothesis, we have validated d-serine degradation as a promising target for disruption of the most common P. mirabilis infections.

## RESULTS

### Proteus mirabilis can utilize d-serine as a sole source of carbon and nitrogen.

**FIG 1:**
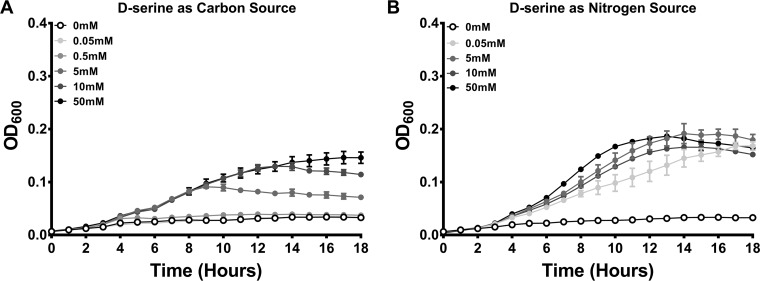
Proteus mirabilis can utilize d-serine as a sole source of carbon and nitrogen. Proteus mirabilis strain HI4320 was cultured in LB broth overnight and diluted 1:100 into *Proteus* minimal salts medium (PMSM) containing increasing concentrations of d-serine as the sole source of carbon (A) or nitrogen (B). Growth was assessed at 37°C with aeration in a 96-well plate reader by measurement of OD_600_ at hourly intervals for 18 h. Error bars represent means ± standard deviations (SDs) from at least 6 replicates, and graphs are representative of 4 independent experiments. The addition of ≥0.5 mM d-serine as a carbon source or ≥0.05 mM d-serine as a nitrogen source promoted significant growth as determined by paired two-way ANOVA with Dunnett’s test for multiple comparisons (*P < *0.001).

Based on prior studies, human urine contains 0.03 to 1 mM d-serine at a 1:1 ratio with l-serine (24–27). Consistent with these studies, our pool of urine from female donors contained 143 ± 17 µM total serine, with 64 ± 3 µM d-serine and 79 ± 14 µM l-serine as assessed by a fluorometric dl serine assay. We therefore generated a *Proteus* minimal salts medium (PMSM) with ≥50 µM d-serine as a sole source of carbon (Fig. 1A) or nitrogen (Fig. 1B) and assessed the growth of P. mirabilis strain HI4320. Concentrations as low as 50 µM supported growth of P. mirabilis when d-serine was supplied as the nitrogen source, while 5 mM was necessary for growth as a sole carbon source, and 10 mM supported optimal growth under both conditions. Thus, P. mirabilis is capable of utilizing d-serine as a sole carbon or nitrogen source, and subsequent experiments utilized a final concentration of 10 mM.

### d-Serine utilization requires *dsdA, dsdX,* and *dsdC*.

**FIG 2:**
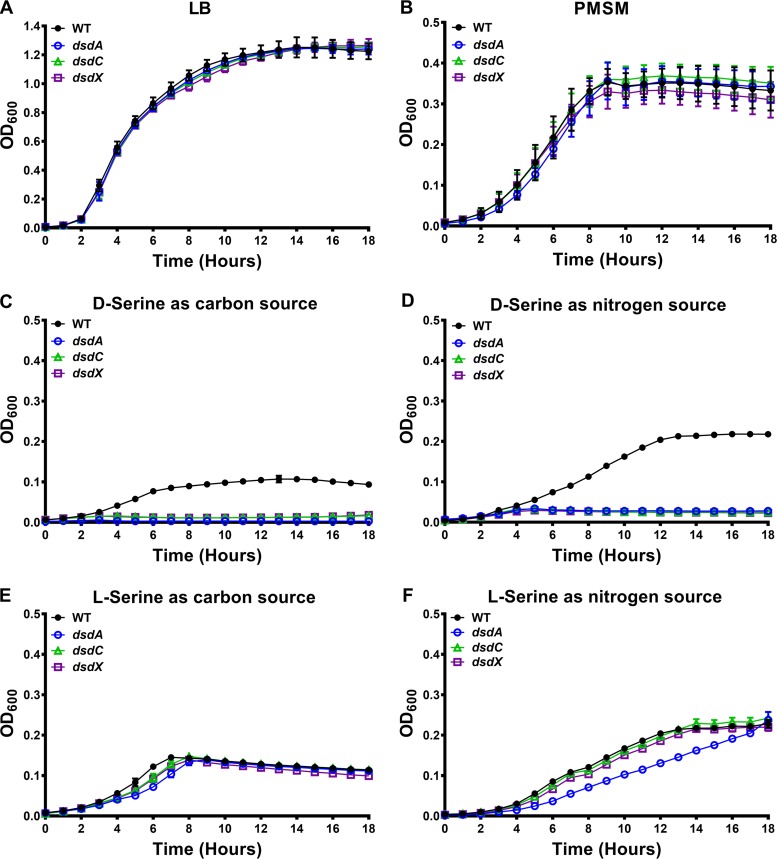
Proteus mirabilis strain HI4320 (WT) and isogenic mutants for *dsdA*, *dsdC*, or *dsdX* were cultured in LB broth overnight and diluted 1:100 into fresh LB (A), PMSM (B), PMSM containing 10 mM d-serine as the sole source of carbon (C), PMSM containing 10 mM d-serine as the sole source of nitrogen (D), PMSM containing 10 mM l-serine as the sole source of carbon (E), or PMSM containing 10 mM l-serine as the sole source of nitrogen (F), and growth was assessed as described in the legend for Fig. 1. Error bars represent means ± standard deviations (SDs) from at least 6 replicates, and graphs are representative of 3 independent experiments. In PMSM with d-serine as the sole source of carbon or nitrogen, the WT exhibited significantly increased growth compared to that of the mutants by paired two-way ANOVA with Dunnett’s test for multiple comparisons (*P < *0.001).

Three isogenic mutants were generated in P. mirabilis strain HI4320 to disrupt d-serine import (*dsdX*), d-serine degradation (*dsdA*), and the divergently transcribed activator of the d-serine degradation operon (*dsdC*) individually. To begin characterizing the role of these genes in d-serine utilization, we first assessed growth of wild-type (WT) P. mirabilis and the three mutants in LB broth (Fig. 2A) and PMSM (Fig. 2B). All three mutants grew similarly to the WT, indicating that disruption of the operon does not affect growth under standard laboratory growth conditions with other carbon and nitrogen sources. When the formulation of PMSM was modified to use 10 mM d-serine as the sole source of carbon (Fig. 2C) or nitrogen (Fig. 2D), only the WT strain was capable of growing, indicating that transcriptional activation, import, and degradation are all needed for d-serine utilization. This observation was confirmed by directly assessing viable CFU of the WT and the *dsdA* mutant during incubation in PMSM with 10 mM d-serine as a sole source of carbon or nitrogen (see Fig. S1 in the supplemental material). Notably, the defect was specific for d-serine, as all three mutants exhibited comparable growth to that of the WT P. mirabilis in PMSM with 10 mM l-serine as a sole source of carbon (Fig. 2E) or nitrogen (Fig. 2F). Furthermore, the growth defect of the *dsdA* mutant was partially complemented by expression of the WT gene from a plasmid (see Fig. S2). Taken together, the *dsdXA* operon is specific for d-serine utilization in P. mirabilis and dispensable for l-serine. This finding is in agreement with the presence of separate genes for l-serine degradation in P. mirabilis (*sdaA*, *sdaB*, and the l-serine transporter, *sdaC*) (29).

10.1128/mSphere.00020-19.6 FIG S1 *dsdA* is required for growth of P. mirabilis when d-serine is the sole carbon or nitrogen source. Proteus mirabilis strain HI4320 (WT) and the *dsdA* mutant were cultured in LB broth overnight, diluted 1:100 into PMSM with 10 mM d-serine as the sole source of carbon or nitrogen, and incubated at 37°C with aeration for 24 h. An aliquot was taken at the start of the culture, at 3 h, and at 24 h to assess viability by plating on LB agar for determination of log_10_ CFU/ml. Error bars represent means ± SDs from three independent experiments. Download FIG S1, TIF file, 0.2 MB. Copyright © 2019 Brauer et al. 2019 Brauer et al. This content is distributed under the terms of the Creative Commons Attribution 4.0 International license.

10.1128/mSphere.00020-19.7 FIG S2 d-Serine utilization defects can be partially complemented. Proteus mirabilis strain HI4320 (WT) harboring pGEN-MCS, the *dsdA* mutant harboring pGEN-MCS, and the *dsdA* mutant complemented with the WT *dsdA* gene on pGEN-*dsdA* were cultured in LB broth with ampicillin overnight and diluted 1:100 into PMSM containing 10 mM d-serine as the sole source of carbon (A) or nitrogen (B). Growth was assessed at 37°C in a 96-well plate reader by measurement of OD_600_ at hourly intervals for 18 h. Error bars represent means ± standard deviations (SDs) from two independent experiments with 6 replicates each. The complemented *dsdA* strain exhibited significantly increased growth under both conditions compared to that of the *dsdA* strain harboring pGEN-MCS by paired two-way ANOVA with Dunnett’s test for multiple comparisons (*P < *0.05). However, growth was not complemented to the level observed for the WT. Download FIG S2, TIF file, 0.4 MB. Copyright © 2019 Brauer et al. 2019 Brauer et al. This content is distributed under the terms of the Creative Commons Attribution 4.0 International license.

To investigate the generalizability of d-serine utilization in P. mirabilis, we tested the ability of 14 clinical isolates to utilize d-serine as a sole nitrogen source (see Fig. S3). This condition was chosen as it supported the more robust growth of P. mirabilis than when d-serine was used as a sole carbon source. Despite moderate differences in overall growth rates in PMSM, all fourteen isolates (100%) exhibited robust growth in PMSM with d-serine as the sole nitrogen source, indicating that this feature is not unique to strain HI4320. Conservation of d-serine utilization was further explored using the BLAST service through PATRIC (30). Each protein sequence was queried against all 19 of the currently available complete genome sequences of P. mirabilis. DsdA exhibited 100% identity in 18/19 isolates and 99% identity in the remaining isolate (see Table S1), DsdX exhibited 100% identity in all 19 isolates (see Table S2), and DsdC exhibited 100% identity in 12/19 isolates and 99% identity in the other 7 (data not shown). Thus, d-serine utilization is highly conserved in P. mirabilis.

10.1128/mSphere.00020-19.1 TABLE S1 BLAST comparison of *dsdA* among *P. mirabilis* genomes. Download Table S1, XLSX file, 0.1 MB. Copyright © 2019 Brauer et al. 2019 Brauer et al. This content is distributed under the terms of the Creative Commons Attribution 4.0 International license.

10.1128/mSphere.00020-19.2 TABLE S2 BLAST comparison of *dsdX* among *P. mirabilis* genomes. Download Table S2, XLSX file, 0.1 MB. Copyright © 2019 Brauer et al. 2019 Brauer et al. This content is distributed under the terms of the Creative Commons Attribution 4.0 International license.

10.1128/mSphere.00020-19.8 FIG S3 d-Serine degradation is common among P. mirabilis clinical isolates. Fourteen Proteus mirabilis clinical isolates were cultured in LB broth overnight and diluted 1:100 into PMSM minimal medium containing 10 mM d-serine as the sole source of nitrogen. Growth was assessed at 37°C in a 96-well plate reader by measurement of OD_600_ at hourly intervals for 18 h, and P. mirabilis strain HI4320 and the *dsdA* mutant were included for comparison. Error bars represent means ± standard deviations (SDs) from two independent experiments with 6 replicates each. All fourteen strains were capable of growing on d-serine as the sole source of nitrogen, indicative of d-serine degradation. Download FIG S3, TIF file, 0.6 MB. Copyright © 2019 Brauer et al. 2019 Brauer et al. This content is distributed under the terms of the Creative Commons Attribution 4.0 International license.

### d-Serine impairs growth of P. mirabilis in the presence of a rich carbon source.

**FIG 3:**
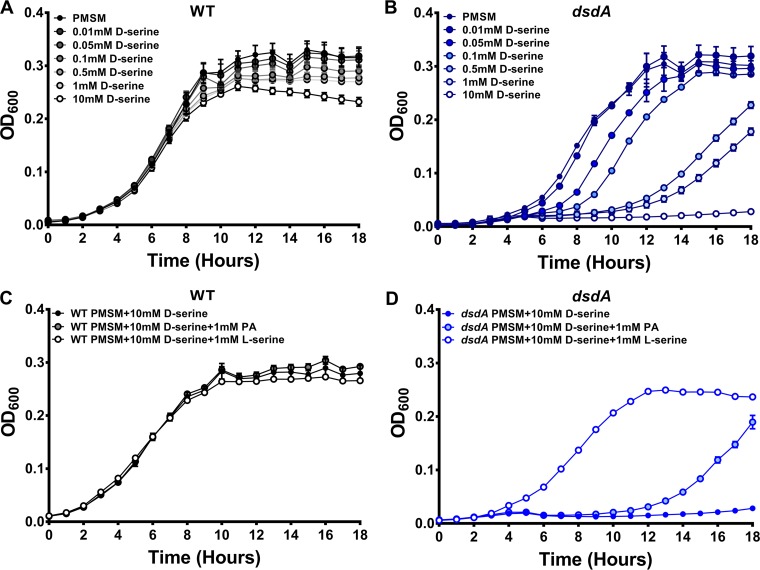
d-Serine toxicity perturbs growth of P. mirabilis in minimal medium with a rich carbon source. Proteus mirabilis strain HI4320 (WT) (A and C) and the *dsdA* mutant (B and D) were cultured in LB broth overnight and diluted 1:100 into the standard formulation of PMSM supplemented with 10 mM d-serine (A and B) or 10 mM d-serine and either 1 mM calcium pantothenic acid or 1 mM l-serine (C and D). Growth was assessed as described in the legend for Fig. 1. Error bars represent means ± standard deviations (SDs) with at least 6 replicates, and graphs are representative of 4 independent experiments. Visible differences in growth curves for the *dsdA* mutant were confirmed by paired two-way ANOVA with Dunnett’s test for multiple comparisons (*P < *0.05).

In E. coli, the presence of d-serine in minimal medium with a rich carbon source has a bacteriostatic effect due to perturbation of l-serine biosynthesis and/or pantothenate (vitamin B_5_) biosynthesis, and *dsdA* mutants are exquisitely susceptible to this toxicity (23). To determine if serine similarly impairs P. mirabilis growth in PMSM with a rich carbon source, the WT and the *dsdA* mutant were cultured in the standard formulation of PMSM supplemented with increasing concentrations of d-serine. Growth of the WT strain was largely unchanged by the addition of up to 10 mM d-serine (Fig. 3A), while growth of the *dsdA* mutant began to be perturbed by as little as 50 µM d-serine and was completely abrogated by the addition of 10 mM d-serine (Fig. 3B). Supplementation of PMSM with 10 mM d-serine also perturbed growth of the *dsdC* and *dsdX* mutants (see Fig. S4A), albeit not to the same extent as *dsdA*, and growth perturbation was specific to d-serine, as l-serine did not impair growth (Fig. S4B). Notably, growth perturbation of the *dsdA* mutant was fully complemented by expression of the WT gene from a plasmid (see Fig. S5).

10.1128/mSphere.00020-19.9 FIG S4 Proteus mirabilis strain HI4320 (WT) and isogenic mutants for *dsdA*, *dsdC*, or *dsdX* were cultured in LB broth overnight and diluted 1:100 into the standard formulation of PMSM supplemented with either 10 mM d-serine (A) or 10 mM l-serine (B). Growth was assessed as described above. Error bars represent means ± standard deviations (SDs) with at least 6 replicates, and graphs are representative of at least 2 experiments. Download FIG S4, TIF file, 0.6 MB. Copyright © 2019 Brauer et al. 2019 Brauer et al. This content is distributed under the terms of the Creative Commons Attribution 4.0 International license.

10.1128/mSphere.00020-19.10 FIG S5 Proteus mirabilis strain HI4320 (WT) harboring pGEN-MCS, the *dsdA* mutant harboring pGEN-MCS, and the *dsdA* mutant complemented with the WT *dsdA* gene on pGEN-*dsdA* were cultured in LB broth with ampicillin overnight and diluted 1:100 into the standard formulation of PMSM supplemented with 10 mM d-serine. Growth was assessed as described above. Error bars represent means ± standard deviations (SDs) from 2 independent experiments with 6 replicates each. Download FIG S5, TIF file, 0.2 MB. Copyright © 2019 Brauer et al. 2019 Brauer et al. This content is distributed under the terms of the Creative Commons Attribution 4.0 International license.

To determine if growth perturbation by d-serine is due to interference of l-serine or pantothenate biosynthesis, we attempted to rescue growth of the *dsdA* mutant by the addition of these factors. A concentration of 1 mM was chosen for each, as this concentration did not impact growth of WT P. mirabilis (Fig. 3B). The addition of 1 mM calcium pantothenic acid (PA) to the *dsdA* mutant supported growth of the mutant after a prolonged lag phase, and 1 mM l-serine fully restored growth of the *dsdA* mutant in PMSM containing 10 mM d-serine (Fig. 3C), indicating that the bacteriostatic property of d-serine in PMSM with glycerol is due to the perturbation of both pathways but may be predominantly due to the perturbation of l-serine biosynthesis.

### P. mirabilis does not require d-serine utilization for growth in urine.

**FIG 4:**
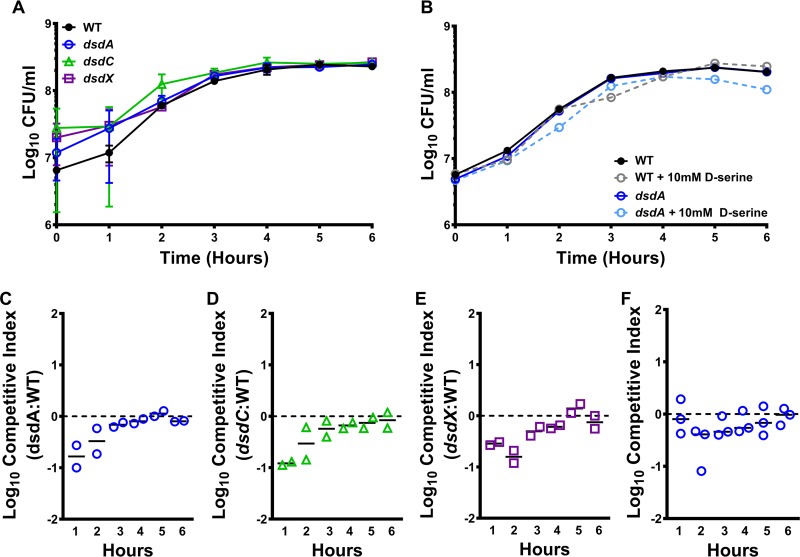
d-Serine does not provide a fitness advantage during growth in urine. Proteus mirabilis strain HI4320 (WT) and isogenic mutants for *dsdA*, *dsdC*, or *dsdX* were cultured in LB broth overnight and diluted 1:100 into pooled filter-sterilized urine from female humans (A to E) or female CBA/J mice (F), and incubated at 37°C with aeration for 6 h. (A) Growth of the WT and each mutant was assessed at hourly intervals by sampling independent urine cultures and plating to determine bacterial CFU/ml. Error bars represent means ± SDs from three independent experiments; no significant differences were identified by two-way ANOVA with multiple-comparison test. (B) The impact of d-serine supplementation on growth was assessed for WT and *dsdA* strains by adding 10 mM d-serine to urine, and growth was assessed as described above. Symbols indicate bacterial CFU/ml for one independent experiment. The relative fitness of *dsdA* (C), *dsdC* (D), and *dsdX* (E) mutants was determined by inoculating urine with a 1:1 mixture of mutant/WT and assessing the CFU of each at hourly intervals. A competitive index (CI) was calculated at each time point by dividing the ratio of mutant/WT for a given time point by the ratio of mutant/WT at time zero. Each symbol represents the log_10_ CI for one independent experiment. (F) The relative fitness of the *dsdA* mutant was also assessed in mouse urine, as above. Each symbol represents the log_10_ CI for one independent experiment, error bars represent the medians, and the dashed lines indicate log_10_ CI of 0 (the expected value if the ratio of mutant/WT is 1:1). No significant differences in CIs were identified by Wilcoxon signed-rank test.

The growth characteristics of P. mirabilis HI4320 and its isogenic d-serine mutants in minimal medium recapitulated what was previously known about this pathway in E. coli. However, the ideal growth medium for exploring the importance of this pathway to CAUTI is urine. We previously determined that P. mirabilis growth in urine is self-limiting in a test tube due to its potent urease enzyme and resulting increase in pH (13). A 6-h time course was therefore utilized to assess the contribution of d-serine degradation to growth in diluted urine, as viability begins to decrease after 6 h. P. mirabilis HI4320 and the three mutants were cultured in dilute human urine, and growth was assessed at hourly intervals by the determination of CFU (Fig. 4A). All three mutants grew comparably to the WT and achieved stationary phase at a similar cell densities. To determine if excess d-serine perturbs growth in urine, the WT and the *dsdA* mutant were cultured in urine with or without the addition of 10 mM d-serine (Fig. 4B). However, no significant differences in growth were observed. Thus, P. mirabilis likely utilizes different carbon and nitrogen sources to support growth in urine over a 6-h time course and does not require d-serine degradation within this time frame. Similarly, it can be inferred that l-serine biosynthesis is also dispensable within this time frame, as this pathway is inhibited in the *dsdA* mutant supplemented with 10 mM d-serine.

Subtle fitness defects can often be magnified when a mutant strain directly competes against a parental isolate. To confirm the conclusions drawn from the independent culture experiments, urine cultures were inoculated with a 1:1 mixture of each mutant versus the WT, and a competitive index was calculated based on the ratio of mutant/WT at the start of the experiment and hourly thereafter (Fig. 4C to E). Despite a slight trend toward decreased fitness from 1 to 2 h postinoculation (hpi), the mutants were not significantly outcompeted by the WT in human urine, confirming that d-serine utilization does not contribute to P. mirabilis growth in human urine *ex vivo* within this 6-h time frame.

Given that the importance of this pathway was initially indicated by infection studies in mice, the competition experiment growth study was repeated for the *dsdA* mutant in urine collected from naive female CBA/J mice (Fig. 4F). Loss of *dsdA* did not result in a fitness defect in mouse urine, confirming that P. mirabilis does not require d-serine utilization or detoxification for growth in mammalian urine *ex vivo* within a 6-h time frame.

### d-Serine utilization does not contribute to motility or urease activity.

**FIG 5:**
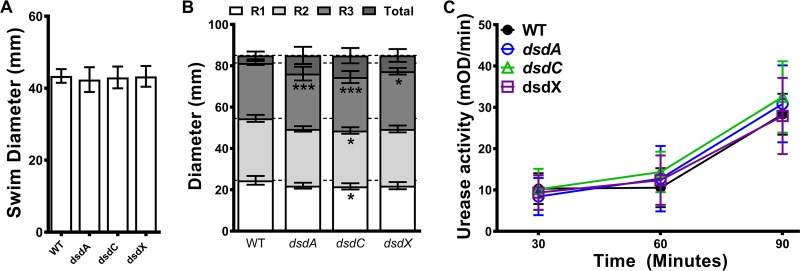
Loss of d-serine utilization does not impact P. mirabilis motility or urease activity. Proteus mirabilis strain HI4320 (WT) and isogenic mutants for *dsdA*, *dsdC*, or *dsdX* were cultured in LB broth overnight and stab inoculated into motility agar (A), inoculated onto the surfaces of swarm agar plates (B), or subcultured in human urine for measurement of urease activity (C). Motility diameters were measured in millimeters after 16 h of incubation at 30°C (A) or 37°C (B); error bars represent means ± SDs from three independent experiments with at least 3 replicates each. *, *P < *0.05, ***, *P < *0.001 by two-way ANOVA with multiple-comparison test. (C) Urease activity was measured at 30-min intervals; error bars represent means ± SDs from three independent experiments. No significant differences were identified by two-way ANOVA.

P. mirabilis is perhaps most well known for two factors that contribute to its ability to cause CAUTI: flagellum-mediated motility and hydrolysis of urea to ammonia and CO_2_ by urease. Prior to conducting infection studies with the d-serine utilization mutants, we first sought to determine if disruption of the *dsdCXA* operon impacts motility or urease activity *in vitro* (Fig. 5). All three mutants were capable of swimming to the same extent as the WT strain (Fig. 5A), and all three swarmed the full diameter of the petri dish similarly to the WT, despite minor decreases in the diameter of each swarm ring (Fig. 5B). Although the decreased diameter of each swarm ring was statistically significant, a decrease in swarm ring diameter of 1 to 3 mm is unlikely to be physiologically relevant or impair fitness. All three mutants also exhibited similar urease activity kinetics compared to those of the WT (Fig. 5C).

### d-Serine utilization provides P. mirabilis with a fitness advantage within the urinary tract.

**FIG 6:**
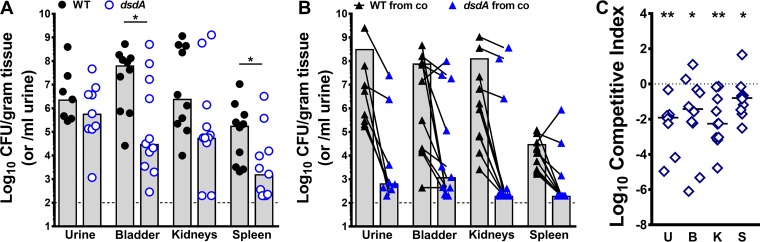
d-Serine utilization contributes to P. mirabilis fitness in a murine model of CAUTI. Proteus mirabilis strain HI4320 (WT) and the *dsdA* mutant were cultured in LB broth overnight, washed once in PBS, and adjusted to 2 × 10^6^ CFU/ml. (A) CBA/J mice were transurethrally inoculated with 50 µl of either WT (*n* = 10) or *dsdA* (*n* = 11) strains at 1 × 10^5^ CFU, and a 4-mm segment of silicone catheter tubing was advanced into the bladder during inoculation. Mice were euthanized 96 h postinoculation (hpi), and bacterial burden was determined in the urine, bladders, kidneys, and spleens. Each symbol represents the log_10_ CFU per milliliter of urine or gram of tissue from an individual mouse, gray bars represent the medians, and the dashed line indicates the limit of detection. *, *P < *0.05 by nonparametric Mann-Whitney test. (B and C) CBA/J mice (*n* = 11) were inoculated as described above with a 1:1 mixture of WT and *dsdA* strains to determine fitness during cochallenge. (B) Each symbol represents the log_10_ CFU per milliliter of urine or gram of tissue from an individual mouse, with the WT and *dsdA* CFU connected by a black line. Gray bars represent the means, and the dashed line indicates the limit of detection. (C) A competitive index was calculated as described above. Each symbol represents the log_10_ CI for an individual mouse, error bars represent the medians and the dashed line indicates log_10_ CI = 0 (the expected value if the ratio of mutant/WT is 1:1). *, *P < *0.05, **, *P < *0.01 by Wilcoxon signed-rank test.

Considering that disruption of *dsdA* resulted in the most pronounced growth phenotypes, this mutant was chosen for experimental infection studies in order to fully explore the contribution of d-serine degradation to P. mirabilis fitness. To assess the contribution of *dsdA* to fitness during CAUTI, female CBA/J mice were transurethrally inoculated with P. mirabilis HI4320, the *dsdA* mutant, or a 1:1 mix. At 96 hpi, mice were euthanized and the bacterial burden was assessed in the urine, catheterized bladders, kidneys, and spleens (Fig. 6). During independent challenge, the *dsdA* mutant was capable of colonizing the urine to a similar level as the WT (*P = *0.3510 by nonparametric Mann-Whitney test), but colonization was reduced in the kidneys (*P = *0.0944) and significantly decreased in the catheterized bladders (*P = *0.0197) and spleens (*P = *0.0355) (Fig. 6A). The colonization defect was even more severe during direct competition with the WT strain, as the *dsdA* mutant was significantly outcompeted by the WT in the urine, bladders, kidneys, and spleens (Fig. 6B and C). Therefore, even though d-serine utilization does not contribute to growth in urine *ex vivo* during a 6-h time course, this capability is critical for survival of P. mirabilis within the urinary tract over the course of 96 h.

### d-Serine utilization provides P. mirabilis with a fitness advantage during polymicrobial infection.

Urine from catheterized individuals frequently contains multiple different bacterial species, particularly when a catheter has been in place for >28 days (5, 11, 31). In our recent investigation of CAUTIs in nursing home residents, P. mirabilis, *Enterococcus* species, and E. coli were the most common uropathogens, and Morganella morganii, *Enterococcus* species, and Providencia stuartii were most prevalent during polymicrobial CAUTIs (5). We therefore sought to determine the importance of P. mirabilis d-serine utilization during coinfection with E. coli, Enterococcus faecalis, M. morganii, and P. stuartii.

Numerous uropathogenic E. coli isolates harbor *dsdA* and are capable of utilizing d-serine as a carbon or nitrogen source, including strain CFT073 (22, 23, 25). However, d-serine utilization has not been explored in the other organisms of interest. Using PATRIC, 44 M. morganii genomes were queried by BLAST against the P. mirabilis *dsdA* amino acid sequence, 35 of which (79%) encoded a *dsdA* homolog with 100% sequence coverage and ≥78% identity (see Table S3). Seven hundred forty-one E. faecalis genomes were similarly queried, and the first 500 hits revealed only one isolate with 99% sequence coverage and 73% amino acid identity, while the rest of the hits had ∼69% coverage and 24% identity due to a diaminopropionate ammonia-lyase (see Table S4). None of the other E. faecalis genomes encode anything annotated as a d-serine dehydratase or ammonium-lyase, and d- and l-serine are listed as extremely poor substrates for diaminopropionate ammonia-lyase. Thus, E. faecalis likely cannot utilize d-serine. Similarly, none of the 16 P. stuartii genome sequences in PATRIC encode a homolog of *dsdA* with greater than 57% sequence coverage and 37% amino acid identity (see Table S5), indicating that this bacterial species likely cannot utilize d-serine as a carbon or nitrogen source.

10.1128/mSphere.00020-19.3 TABLE S3 BLAST comparison of *dsdA* among *M. morganii* genomes. Download Table S3, XLSX file, 0.1 MB. Copyright © 2019 Brauer et al. 2019 Brauer et al. This content is distributed under the terms of the Creative Commons Attribution 4.0 International license.

10.1128/mSphere.00020-19.4 TABLE S4 BLAST comparison of *dsdA* among *E. faecalis* genomes. Download Table S4, XLSX file, 0.1 MB. Copyright © 2019 Brauer et al. 2019 Brauer et al. This content is distributed under the terms of the Creative Commons Attribution 4.0 International license.

10.1128/mSphere.00020-19.5 TABLE S5 BLAST comparison of *dsdA* among *P. stuartii* genomes. Download Table S5, XLSX file, 0.1 MB. Copyright © 2019 Brauer et al. 2019 Brauer et al. This content is distributed under the terms of the Creative Commons Attribution 4.0 International license.

**FIG 7:**
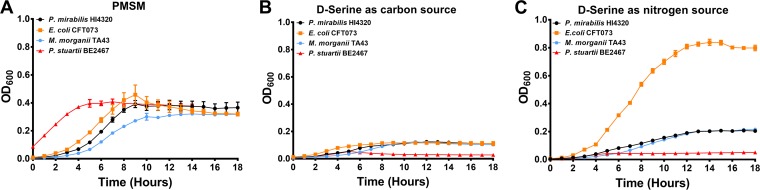
Not all uropathogens can utilize d-serine as a sole source of carbon or nitrogen. Proteus mirabilis strain HI4320 (WT), Escherichia coli strain CFT073, Providencia stuartii strain BE2467, and Morganella morganii strain TA43 were cultured in LB broth overnight and diluted 1:100 into the standard formulation of PMSM (A), PMSM containing 10 mM d-serine as the sole carbon source (B), and PMSM containing 10 mM d-serine as the sole nitrogen source (C). Growth was assessed as described above. Error bars represent means ± standard deviations (SD) from 3 experiments with at least 6 replicates each. Growth of P. stuartii was significantly lower than each of the other species in panels B and C as determined by paired two-way ANOVA with Dunnett’s test for multiple comparisons (*P < *0.05).

To confirm these *in silico* analyses, growth curves were conducted in PMSM. Unfortunately, the ability of E. faecalis strain 3143 to utilize d-serine as a sole source of carbon or nitrogen could not be addressed, as eight amino acids are essential for growth of this species (32), rendering it impossible to generate a minimal medium sufficient for testing this hypothesis. To test the other species, different PMSM formulations were inoculated with E. coli CFT073, P. stuartii BE2467, and M. morganii TA43 for comparison to P. mirabilis HI4320 (Fig. 7). All isolates were capable of robust growth in PMSM with glycerol as a carbon source and ammonium sulfate as a nitrogen source (Fig. 7A), and P. stuartii was the only isolate that did not exhibit growth to any extent when d-serine was supplied as the sole source of carbon (Fig. 7B) or nitrogen (Fig. 7C).

**FIG 8:**
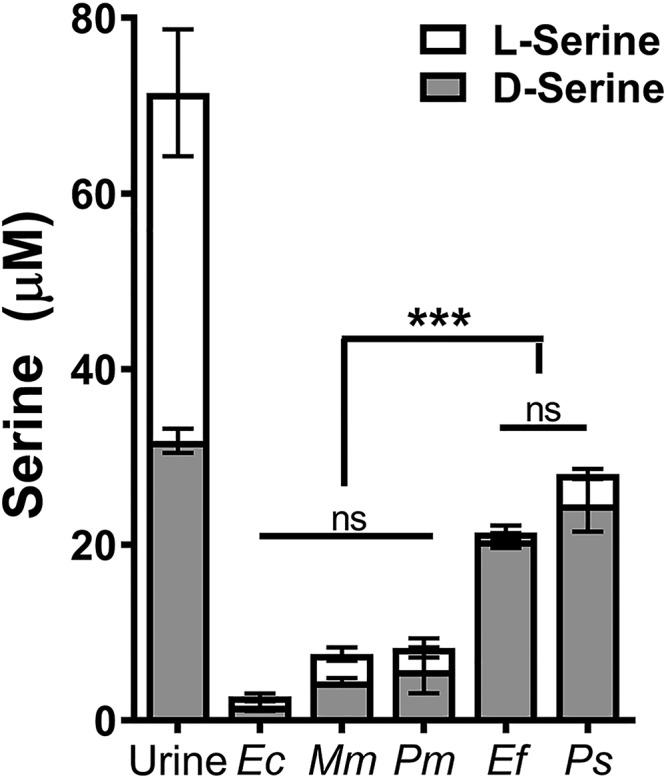
Not all uropathogens degrade d-serine during growth in human urine. Proteus mirabilis strain HI4320 (WT), E. coli CFT073 (*Ec*), M. morganii TA43 (*Mm*), and P. stuartii BE2467 (*Ps*) were cultured in LB broth overnight and E. faecalis 3143 (*Ef*) was cultured in BHI overnight. Overnight cultures were diluted 1:100 into pooled filter-sterilized urine from female donors that had been diluted 1:1 with sterile saline and incubated for 18 h at 37°C with aeration, and d- and l-serine were quantified using a fluorometric detection kit for comparison to the starting urine pool. Bars represent total serine concentration, divided into d-serine and l-serine for three independent replicates. ns, *P *>* *0.05, ***, *P < *0.001 by two-way ANOVA.

We next assessed the ability of each of the uropathogens to deplete serine during an 18-h incubation in dilute human urine (Fig. 8). The 18-h time point was chosen to capture maximal serine depletion. All five uropathogens significantly depleted serine from urine, with the greatest depletion by E. coli, M. morganii, and P. mirabilis. Notably, all five uropathogens depleted 90% to 99% of l-serine in urine, while only E. coli, M. morganii, and P. mirabilis significantly depleted d-serine. Taken together, these data indicate that all five uropathogens are capable of utilizing l-serine, but only P. mirabilis, E. coli, and M. morganii can utilize d-serine while P. stuartii and E. faecalis cannot.

**FIG 9:**
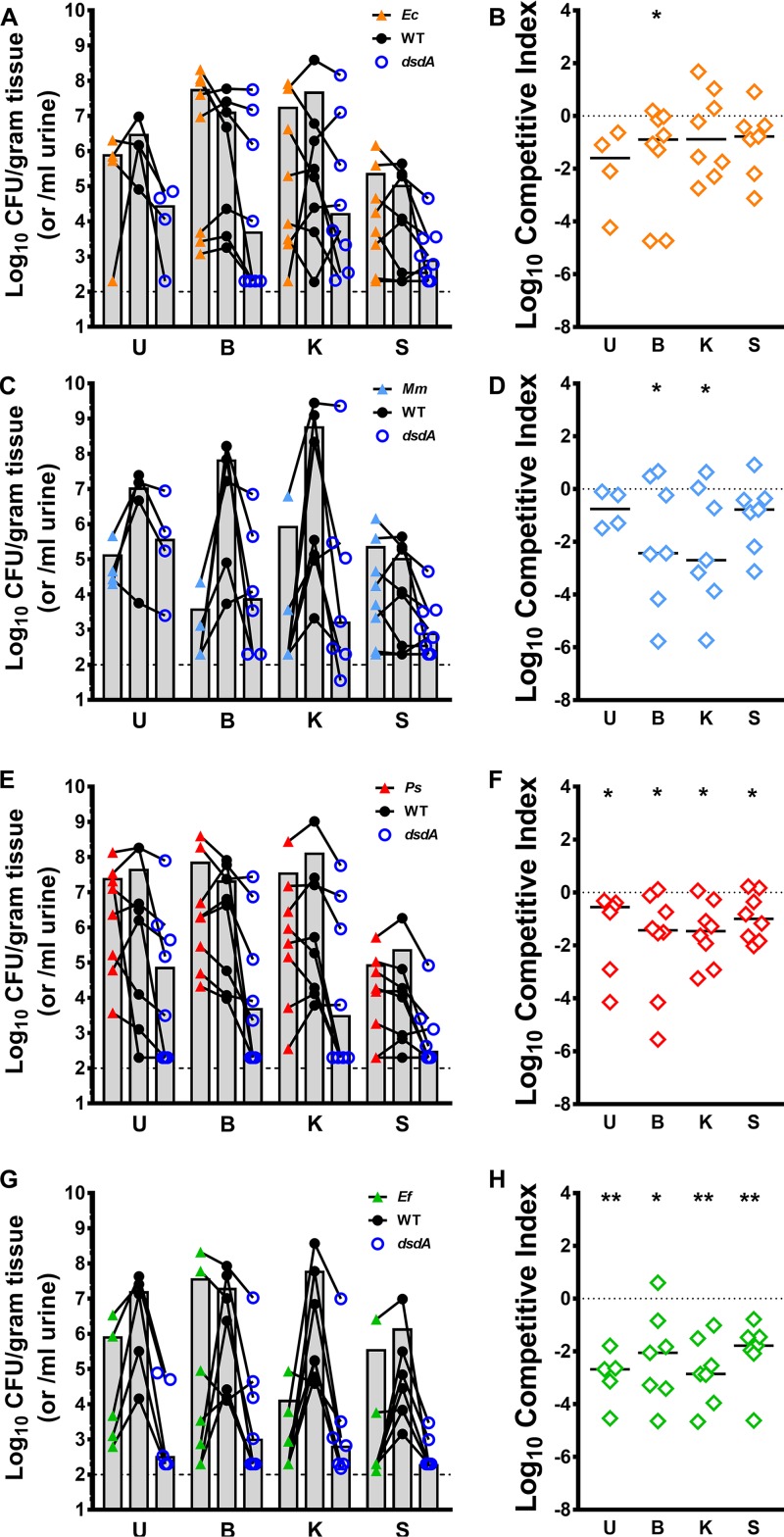
d-Serine utilization provides a fitness advantage to P. mirabilis during polymicrobial CAUTI. Proteus mirabilis strain HI4320 (WT), the *dsdA* mutant (*dsdA*), E. coli CFT073 (*Ec*), P. stuartii BE2467 (*Ps*), and M. morganii TA43 (*Mm*) were cultured in LB broth overnight, and E. faecalis 3143 (*Ef*) was cultured in BHI overnight. All cultures were washed once in PBS and adjusted to 2 × 10^6^ CFU/ml. CBA/J mice were transurethrally inoculated with 50 µl of a mixture containing 5 × 10^4^ CFU of a 1:1 mixture of *dsdA* and WT P. mirabilis and 5 × 10^4^ CFU of either E. coli CFT073 (A and B,; *n* = 8), M. morganii TA43 (C and D; *n* = 7), P. stuartii BE2467 (E and F; *n* = 8), or E. faecalis 3143 (G and H; *n* = 7). A 4-mm segment of silicone catheter tubing was advanced into the bladder during inoculation, and mice were euthanized 96 h postinoculation (hpi) for determination of bacterial burden in the urine, bladders, kidneys, and spleens. (A, C, E, and G) Each symbol represents the log_10_ CFU per milliliter of urine or gram of tissue recovered from an individual mouse, connected by black lines: triangles represent the coinfecting species, black circles represent the WT P. mirabilis, and open blue circles represent the *dsdA* mutant. Gray bars represent the means, and the dashed lines indicate the limits of detection. (B, D, F, and H) Each symbol represents the log_10_ CI for the *dsdA* mutant recovered from an individual mouse for which CFU were above the limit of detection, error bars represent the medians and the dashed lines indicate log_10_ CI = 0 (the expected value if the ratio of mutant/WT is 1:1). *, *P < *0.05, **, *P < *0.01 by Wilcoxon signed-rank test.

Using this collection of uropathogens, we assessed the contribution of d-serine utilization to P. mirabilis fitness during polymicrobial CAUTI with two organisms capable of utilizing d-serine (E. coli and M. morganii) compared to two that are not (P. stuartii and E. faecalis). To establish polymicrobial infections and assess the importance of *dsdA*, CBA/J mice were transurethrally inoculated with 1 × 10^5^ CFU of the following mixture: 5 × 10^4^ CFU of a 1:1 mixture of the *dsdA* mutant and wild-type P. mirabilis and 5 × 10^4^ CFU of the other species of interest. Mice were euthanized at 96 hpi as before to determine bacterial burden in the urine, catheterized bladders, kidneys, and spleens via CFU (Fig. 9A, C, E, and G), and a competitive index was calculated using the ratio of the *dsdA* mutant to WT P. mirabilis recovered from each organ (Fig. 9B, D, F, and H). The mutant/WT cochallenge during coinfection strategy was used for these experiments to control for potential differences in inflammation, tissue damage, and colonization level during each coinfection, allowing for a direct assessment of the relative fitness of the *dsdA* mutant compared to that of its parental isolate. All mice exhibited cocolonization of P. mirabilis and the other uropathogen of interest, indicating that polymicrobial infections were established. Interestingly, the *dsdA* mutant was significantly outcompeted by WT P. mirabilis in at least one organ during all four coinfections, even when cocolonizing with another organism capable of degrading d-serine, indicating that d-serine utilization provides P. mirabilis with a competitive advantage during polymicrobial CAUTI with each of the most common cocolonizing organisms. However, it is notable that the *dsdA* mutant had the most statistically significant fitness defects during coinfection with P. stuartii and E. faecalis, the two uropathogens that do not degrade d-serine.

### d-Serine utilization provides P. mirabilis with a fitness advantage within the bloodstream.

CAUTI is the most common cause of secondary bacteremia in health care facilities and is a leading cause of mortality in catheterized patients (11, 33–35). However, the contribution of P. mirabilis fitness factors to survival within the bloodstream is rarely assessed. Notably, it was previously shown in mice and humans that kidney injury alters the distribution of d-serine, leading to a decrease in urine and an increase in serum (36–38). It is therefore likely that kidney damage during infection could result in greater serum d-serine concentrations, which may promote survival of d-serine-degrading bacteria within the bloodstream by providing an additional nutrient source. Considering that d-serine concentrations are the highest in urine and serum and the P. mirabilis *dsdA* mutant exhibited a significant decrease in spleen colonization during independent challenge, cochallenge, and coinfection, we hypothesized that d-serine utilization may provide P. mirabilis with an advantage during dissemination from the urinary tract as well as during direct inoculation into the bloodstream.

**FIG 10:**
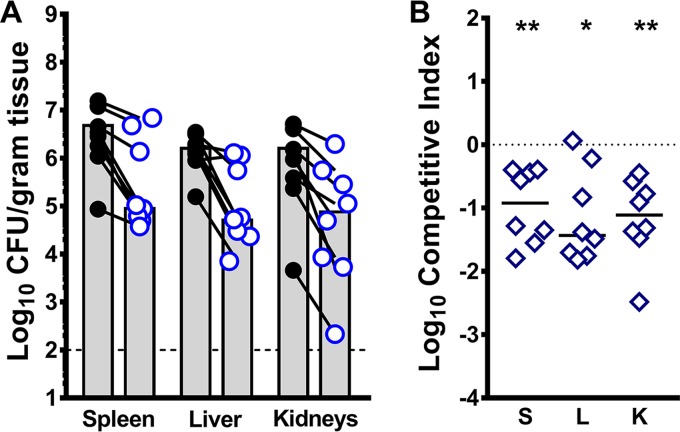
d-Serine utilization contributes to fitness of P. mirabilis within the bloodstream. Proteus mirabilis strain HI4320 (WT, black circles) and the *dsdA* mutant (open blue circles) were cultured in LB broth overnight, washed once in PBS, and adjusted to 2 × 10^8^ CFU/ml. CBA/J mice (*n* = 8) were inoculated by tail vein injection of 100 µl containing a 1:1 mixture of *dsdA* and WT strains. Mice were euthanized at 24 hpi, and bacterial burden was determined in the spleens, livers, and kidneys. (A) Each symbol represents the log_10_ CFU per gram of tissue from an individual mouse, with the WT and *dsdA* CFU connected by black lines. Gray bars represent the means, and the dashed line indicates the limit of detection. (B) A competitive index was calculated as described above. Each symbol represents the log_10_ CI for an individual mouse, error bars represent the medians, and the dashed line indicates log_10_ CI = 0 (the expected value if the ratio of mutant/WT is 1:1). *, *P < *0.05, **, *P < *0.01 by Wilcoxon signed-rank test.

To test this hypothesis, bacteremia was established in CBA/J mice via tail vein injection of 1 × 10^7^ CFU of a 1:1 mix of P. mirabilis HI4320 and the *dsdA* mutant. Mice were euthanized after 24 h, and the spleens, livers, and kidneys were harvested for determination of bacterial burden (Fig. 10). In all three organs, the *dsdA* mutant was outcompeted by the WT approximately 10- to 50-fold, indicating that d-serine utilization provides P. mirabilis with a fitness advantage within the bloodstream in addition to contributing to urinary tract colonization and dissemination to the bloodstream. In conclusion, d-serine utilization represents an important fitness factor for the ability of P. mirabilis to establish infection within the urinary tract and bloodstream, regardless of the presence of other organisms. d-Serine uptake and degradation may therefore be a promising target for disrupting P. mirabilis infections.

## DISCUSSION

Approximately half of all individuals catheterized for ≥7 days will develop catheter-associated bacteriuria, and the majority of individuals catheterized long-term will experience at least one episode of CAUTI (11), making CAUTI one of the most common health care-associated infections (11). CAUTI is also the most common cause of secondary bacteremia in health care facilities, with a 1-year mortality rate of 10% to 13% in most settings and can be as high as 66% (11, 33–35). The Gram-negative bacterium Proteus mirabilis has been identified as a leading cause of both CAUTI (2–5) and secondary bacteremia (9, 39–44), particularly in nursing homes. There are no licensed vaccines against P. mirabilis, and antibiotic-resistant isolates are on the rise, including carbapenem-resistant isolates (45–49). Thus, it is imperative to identify novel targets for the prevention or disruption of infections caused by this organism. In this study, we show that P. mirabilis can utilize d-serine as a sole carbon or nitrogen source, that this capability is highly conserved among P. mirabilis isolates from diverse sources, and that d-serine utilization is an infection-specific fitness factor for P. mirabilis colonization of the catheterized urinary tract as well as the bloodstream. Thus, d-serine import and degradation may be novel targets for preventing or disrupting P. mirabilis colonization.

Considering that P. mirabilis is one of the most common organisms present during polymicrobial CAUTI (2, 5, 11, 12), and secondary bacteremia is also frequently polymicrobial (10, 11), an additional consideration in identifying novel targets for therapeutic intervention is the relevance of the target during polymicrobial infection. In our recent investigation of CAUTIs in nursing home residents, the three most common organisms were P. mirabilis, *Enterococcus* species, and E. coli, and we observed a trend toward certain organisms being more prevalent during polymicrobial CAUTI than as the sole causative agent, specifically, Morganella morganii (71% polymicrobial), *Enterococcus* species (60% polymicrobial), and Providencia stuartii (57% polymicrobial) (5). We therefore investigated d-serine utilization in these other uropathogens, and determined that E. coli and M. morganii are also capable of utilizing d-serine as a sole carbon or nitrogen source and deplete d-serine during incubation in human urine, while P. stuartii and E. faecalis cannot. Despite these differences, d-serine utilization remained an important fitness factor for P. mirabilis during coinfection with any of these four species. Thus, targeting d-serine import and degradation may be an effective method for preventing or disrupting P. mirabilis colonization during polymicrobial infection in addition to single-species infection.

d-Serine utilization has been explored in other uropathogens. For instance, Pseudomonas aeruginosa harboring *dsdA* can use d-serine as a nitrogen source but not as a carbon source (20), S. saprophyticus harboring *dsdA* can utilize d-serine as a carbon source (21), and E. coli harboring *dsdA* can utilize d-serine as either a carbon or nitrogen source (22). Our research builds on these findings by revealing that P. mirabilis, E. coli, M. morganii, P. stuartii, and E. faecalis all deplete l-serine during growth in human urine *in vitro*, but only P. mirabilis, E. coli, and M. morganii deplete d-serine. Consistent with this observation, only P. mirabilis, E. coli, and M. morganii are capable of utilizing d-serine as a sole carbon or nitrogen source in minimal medium *in vitro*. Notably, the human urine used for our experimental studies contained 143 ± 17 µM total serine, with 64 ± 3 µM d-serine and 79 ± 14 µM l-serine as assessed by a fluorometric dl serine assay, while our minimal medium experiments predominantly utilized 10 mM d- or l-serine. This concentration of serine was chosen for minimal medium experiments as it permitted optimal growth of P. mirabilis when supplied as either the sole carbon or nitrogen source. While 10 mM is ∼150 times the concentration of d-serine in our human urine pool, urine contains numerous additional amino acids and other carbon and nitrogen sources, while the minimal medium recipe only contains serine. Thus, a higher concentration is needed to support growth of P. mirabilis. Despite this limitation, the physiologically relevant concentration of 50 µM d-serine was sufficient to support detectable growth of P. mirabilis when supplied as the nitrogen source, indicating that this concentration could provide P. mirabilis with a fitness advantage during infection.

Interestingly, loss of d-serine degradation results in a modest growth decrease for E. coli CFT073 from 2 to 5 h during incubation in human urine, but this pathway does not provide a significant fitness advantage to E. coli during experimental UTI (25, 50). We show the opposite results for P. mirabilis, in which d-serine degradation does impact growth in human urine *in vitro* over the course of 6 h, but significantly impacts fitness with the mouse urinary tract over 96 h. The discrepancy in the importance of d-serine utilization to growth in human urine *in vitro* likely pertains to the fact that urine contains numerous amino acids and other carbon and nitrogen sources in addition to serine, and the concentrations of these nutrients can differ significantly between urine pools. It is likely that P. mirabilis and E. coli each have a hierarchy of preferred nutrient sources, and they only utilize d-serine after other more favorable nutrients are depleted. E. coli may therefore utilize d-serine in a shorter time frame than P. mirabilis, which would explain the difference in *dsdA* requirements for E. coli and P. mirabilis during short-term growth in urine. However, P. mirabilis clearly benefits from the use of the amino acid during infection, as the *dsdA* mutant exhibits dramatic fitness defects relative to the WT.

A prior study showed that E. coli isolates have very different gene expression profiles during growth in urine in a test tube *in vitro* compared to that within a host during infection (51), and so the differential requirement for d-serine degradation in urine and within the mouse urinary tract is not unexpected. The difference in *in vivo* d-serine utilization between E. coli and P. mirabilis may also relate to differences in the metabolic pathways utilized by these species during UTI. For instance, prior work has indicated that E. coli CFT073 favors gluconeogenesis during experimental UTI, while P. mirabilis favors glycolysis and catabolism of peptides, amino acids, and pyruvate catabolism (19, 52). d-Serine utilization may therefore be a low priority or back-up nutrient for E. coli within the urinary tract, while it plays a more detectable role in urine *ex vivo.* P. mirabilis also possesses two distinctive metabolic features that set it apart from most E. coli isolates*:* the ability to use citrate as a sole carbon source and the ability to utilize urea for production of an abundant nitrogen source via the urease enzyme (1). Considering that citrate and urea are both abundant in urine, P. mirabilis likely sees the urinary tract as a very different environment than other organisms with respect to the carbon/nitrogen ratio. It is therefore not surprising that it would utilize different metabolic pathways, particularly ones that may provide a fitness advantage in a polymicrobial setting. In agreement with this hypothesis, Staphylococcus saprophyticus is another urease-producing organism (53), and d-serine degradation contributes to S. saprophyticus fitness within the bladder and kidney during experimental UTI (21).

It is also notable that P. mirabilis can utilize l-serine as a sole source of carbon or nitrogen, and two genes involved in l-serine degradation (*sdaA* and *sdaB*) were previously identified as fitness factors during CAUTI (19). The ratio of d-serine to l-serine is approximately 1:1 in urine and 1:100 in serum (26, 27), indicating that l-serine degradation could also provide a viable strategy for growth in urine. This hypothesis is supported by the finding that P. mirabilis fully depletes l-serine as well as d-serine during incubation in dilute human urine. It is therefore likely the P. mirabilis degrades both d-serine and l-serine within the catheterized urinary tract and that d-serine degradation most likely contributes to P. mirabilis metabolism in this niche rather than being required for detoxification of l-serine or pantothenate biosynthesis. However, the relative importance of d-serine versus l-serine degradation remains to be investigated.

In summary, we have characterized serine utilization in five uropathogens and demonstrated d-serine utilization as an important fitness factor for P. mirabilis, regardless of cocolonizing organism and infection model. The d-serine utilization pathway may therefore represent a promising target for prevention or therapeutic intervention. Ongoing efforts are focused on identifying other “core” fitness factors that similarly contribute to P. mirabilis colonization and pathogenicity in multiple infection models and during polymicrobial infection.

## MATERIALS AND METHODS

### Ethics statement.

All animal protocols were approved by the Institutional Animal Care and Use Committee (IACUC) at the State University of New York at Buffalo, Jacobs School of Medicine and Biomedical Sciences (MIC31107Y), and were in accordance with the Office of Laboratory Animal Welfare (OLAW), the United States Department of Agriculture (USDA), and the guidelines specified by the Association for Assessment and Accreditation of Laboratory Animal Care, International (AAALAC, Intl.).

### Bacterial strains and culture conditions.

The clinical isolates utilized in this study were obtained from existing strain libraries and were all collected directly from urine or from the periurethral area. Proteus mirabilis strains include 8 isolates from the urine of catheterized nursing home residents (strains HI4320, AK1391, BA6163, DI120, EL102, HU1069, 936, and 3143) and seven isolates from the periurethral area of catheterized nursing home residents (strains 1161, 1697, 2080, 3318, 3360, 3821, and 4081). Providencia stuartii strain BE2467 and Morganella morganii strain TA43 were isolated from the same cohort of residents as the first six P. mirabilis strains above (3, 54). Enterococcus faecalis strain 3143 was isolated from the same cohort of nursing home residents as P. mirabilis strains 936 and 3143 (5, 14). Escherichia coli strain CFT073 was isolated from the urine of a patient with pyelonephritis (55).

The P. mirabilis d-serine degradation mutants were constructed in strain HI4320 by the insertion of a kanamycin resistance cassette into *dsdA*, *dsdC*, or *dsdX* according to the Sigma TargeTron group II intron protocol, as previously described (56). Mutants were verified by selection on kanamycin and PCR verification. Insertion of a kanamycin cassette via TargeTron retrohoming can result in polar effects. The *dsdA* mutant was therefore complemented by PCR amplifying the WT *dsdA* gene and 500 bp of flanking sequences, ligating to a linearized pGEN-MCS vector, electroporation, and selection on ampicillin.

Bacteria were routinely cultured at 37°C with aeration in 5 ml LB broth (10 g/liter tryptone, 5 g/liter yeast extract, 0.5 g/liter NaCl) or brain heart infusion broth (BHI), or on LB and BHI solidified with 15 g/liter (1.5%) agar. Swarming motility was assessed using swarm agar (LB agar with 5 g/liter NaCl). Swimming motility was assessed using Mot medium (10 g/liter tryptone, 5 g/liter NaCl) solidified with 3 g/liter (0.3%) agar. Proteus mirabilis minimal salts medium (PMSM) (21) was used for studies requiring defined medium [10.5 g/liter K_2_HPO_4_, 4.5 g/liter KH_2_PO_4_, 0.47 g/liter sodium citrate, and 1 g/liter (NH4)_2_SO_4_ supplemented with 0.001% nicotinic acid, 1 mM MgSO_4_, and 0.2% glycerol]. Where indicated, the PMSM formulation was modified by replacing the glycerol or ammonium sulfate with 10 mM d- or l-serine or by adding d- or l-serine or calcium pantothenic acid. Media were supplemented with kanamycin (25 µg/ml), ampicillin (25 µg/ml), chloramphenicol (20 µg/ml), streptomycin (100 µg/ml), or tetracycline (2.5 µg/ml) as needed to differentiate between bacterial strains and species.

### Serine measurement in urine.

Measurement of total serine concentration and assessment of d- versus l-enantiomers were conducted using a fluorometric dl-Serine assay kit (BioVision). Briefly, pooled, filter-sterilized human urine from female donors (Cone Bioproducts) was deproteinized using the sample cleanup mix provided in the kit, filtered through a 10-kDa-molecular-weight-cutoff spin column, and assayed in triplicates according to the manufacturer’s protocol. Serine depletion by P. mirabilis, E. coli, M. morganii, E. faecalis, and P. stuartii was assessed using the same method after an 18-h incubation in urine that had been diluted 1:1 with sterile saline (specific gravity, ∼1.005).

### Growth curves and urine competition experiments.

Overnight cultures of bacterial strains of interest were diluted 1:100 in growth medium (LB or various formulations of PMSM) and distributed into at least 3 wells each of a clear 96-well plate. Plates were incubated at 37°C with continuous double-orbital shaking in a BioTek Synergy H1 96-well plate reader, with a 1°C temperature differential between the top and bottom of the plate to prevent condensation. Bacterial growth was assessed by measuring the optical density at 600 nm (OD_600_) at hourly intervals for a duration of 18 h. Urine growth curves were obtained by diluting overnight cultures of WT bacteria or d-serine utilization mutants 1:100 into pooled, filter-sterilized human urine from female donors (Cone Bioproducts) diluted 1:1 with sterile saline (specific gravity, ∼1.005) or pooled, filter-sterilized mouse urine collected from naive 6- to 8-week-old female CBA/J mice and diluted with sterile saline to a similar specific gravity as human urine. Urine cultures were sampled hourly for the determination of bacterial CFU by plating onto LB agar. The fitness of d-serine utilization mutants during growth in urine was also assessed by direct competition with WT P. mirabilis HI4320. Urine samples were inoculated with a 1:1 mixture of a mutant of interest and the WT, and cultures were sampled hourly for the determination of bacterial CFU by plating onto LB agar (total CFU) and LB with kanamycin (CFU of mutant strain). A competitive index (CI) was calculated as CI = (*dsdA* output/WT output)/(*dsdA* input/WT input).

A log_10_ CI of 0 indicates that the ratio of the strains in the output is similar to that in the input, and neither strain had an advantage, a log_10_ CI of >0 indicates that that the mutant has a competitive advantage over the WT, and a log_10_ CI of <0 indicates that that the mutant is outcompeted by the WT.

### Motility assays.

Swimming motility agar plates (MOT; 10 g/liter tryptone, 0.5 g/liter NaCl, 3 g/liter agar) were stab inoculated with an overnight culture of P. mirabilis HI4320 or isogenic mutant and incubated without inverting at 30°C for 18 h prior to the measurement of swimming diameter. Swarm agar refers to LB agar containing 5 g/liter NaCl. Swarming was assessed by inoculating 5 µl of an overnight culture of P. mirabilis HI4320 or isogenic mutant onto the surface of a swarm plate, allowing the inoculum to soak in for ∼10 min, and incubating at 37°C for 18 h prior to the measurement of the diameter of each swarm ring.

### Urease assay.

Urease activity was measured as described previously (14). Briefly, bacteria were cultured in LB to mid-log phase (OD_600_ of 0.5), pelleted by centrifugation, resuspended in pooled, filter-sterilized dilute human urine (Cone Bioproducts), and incubated at 37°C with aeration. At 30-min intervals, 1 ml was removed from each culture, centrifuged to pellet, and resuspended in 1/10 volume of 0.9% saline. Wells of a 96-well plate containing urine, 0.001% (wt/vol) phenol red, and 250 mM urea were inoculated with 20 μl of the saline resuspension. The OD_562_ was measured using a BioTek Synergy HI over a 10-min kinetic read, and urease activity was expressed as the mean change in optical density per minute (mOD/min), as calculated by the Gen5 software (BioTek).

### Mouse model of CAUTI.

CAUTI studies were carried out as previously described (19). Briefly, the inoculum was prepared by washing overnight cultures of P. mirabilis and the *dsdA* mutant in phosphate-buffered saline (PBS; 0.128 M NaCl, 0.0027 M KCl, pH 7.4), adjusting them to an OD_600_ of 0.2 (∼2 × 10^8^ CFU/ml), and diluting 1:100 to achieve an inoculum of 2 × 10^6^ CFU/ml. Female CBA/J mice aged 6 to 8 weeks (Jackson Laboratory) were anesthetized with a weight-appropriate dose (0.1 ml for a mouse weighing 20 g) of ketamine-xylazine (80 to 120 mg/kg ketamine and 5 to 10 mg/kg xylazine) by intraperitoneal (i.p.) injection and inoculated transurethrally with 50 µl of the diluted suspension (1 × 10^5^ CFU/mouse). A 4-mm segment of sterile silicone tubing (0.64-mm outside diameter [o.d.], 0.30-mm inside diameter [i.d.]; Braintree Scientific, Inc.) was carefully advanced into the bladder during inoculation and retained for the duration of the study as described previously (14, 57). At 96 hpi, urine was collected, the mice were euthanized, and bladders, kidneys, and spleens were harvested into 5-ml Eppendorf tubes containing 1 ml PBS. Tissues were homogenized using a Bullet Blender 5 Gold (Next Advance) and plated using an EddyJet 2 spiral plater (Neutec Group) for determination of CFU using a ProtoCOL 3 automated colony counter (Synbiosis).

For cochallenge experiments, mice were inoculated with a 1:1 mix of WT P. mirabilis and the *dsdA* mutant, and samples were plated onto plain LB agar (total CFU) and LB with kanamycin (*dsdA* CFU). For coinfection experiments utilizing other uropathogens, mice were inoculated with a mixture containing 5 × 10^4^ CFU of a 1:1 mixture of the *dsdA* mutant and WT P. mirabilis and 5 × 10^4^ CFU of the other species, and samples were plated as follows. (i) P. stuartii coinfection samples were plated on plain LB (total CFU), LB with kanamycin (P. stuartii plus *dsdA* CFU), and LB with chloramphenicol (P. stuartii CFU); (ii) E. coli coinfection samples were plated on plain LB (total CFU), LB with kanamycin (*dsdA* CFU), and LB with tetracycline (WT P. mirabilis plus *dsdA* CFU); (iii) E. faecalis coinfection samples were plated on plain LB (total CFU), LB with kanamycin (*dsdA* plus E. faecalis CFU), and BHI with streptomycin (E. faecalis CFU); (iv) M. morganii coinfection samples were plated on plain LB (total CFU), LB with kanamycin (*dsdA* plus M. morganii CFU), and LB with ampicillin (M. morganii CFU).

### Mouse model of bacteremia.

Bacteremia studies were carried out as previously described (58). Briefly, the inoculum was prepared by washing overnight cultures of P. mirabilis HI4320 and the *dsdA* mutant in PBS and diluting to an OD_600_ of 0.1 (∼1 × 10^8^ CFU/ml). Female CBA/J mice aged 6 to 8 weeks (Jackson Laboratory) were inoculated by tail vein injection of 100 µl of a 1:1 mixture of the P. mirabilis HI4320 and *dsdA* mutant suspensions. Mice were euthanized 24 hpi, and organs were harvested into 5 ml Eppendorf tubes containing PBS (1 ml for spleens and kidneys, 2 ml for livers). Tissues were homogenized using a Bullet Blender 5 Gold (Next Advance) and plated onto plain LB agar (total CFU) and LB with kanamycin (*dsdA* CFU) using an EddyJet 2 spiral plater (Neutec Group) for determination of CFU using a ProtoCOL 3 automated colony counter (Synbiosis).

### Statistical analysis.

For motility, urease activity, growth curves, and CFU data, significance was assessed using two-way analyses of variance (ANOVAs) corrected for multiple comparisons. Competitive indices from cochallenge experiments were assessed by Wilcoxon signed-rank tests. These analyses were performed using GraphPad Prism, version 7.03 (GraphPad Software). All *P* values are two-tailed at a 95% confidence interval.
