# Supplementary figures and images for: d-Serine Degradation by Proteus mirabilis Contributes to Fitness during Single-Species and Polymicrobial Catheter-Associated Urinary Tract Infection
Source: mSphere. 2019 Feb 27;4(1):e00020-19. doi: 10.1128/mSphere.00020-19 (PMC6393727; doi:10.1128/mSphere.00020-19)

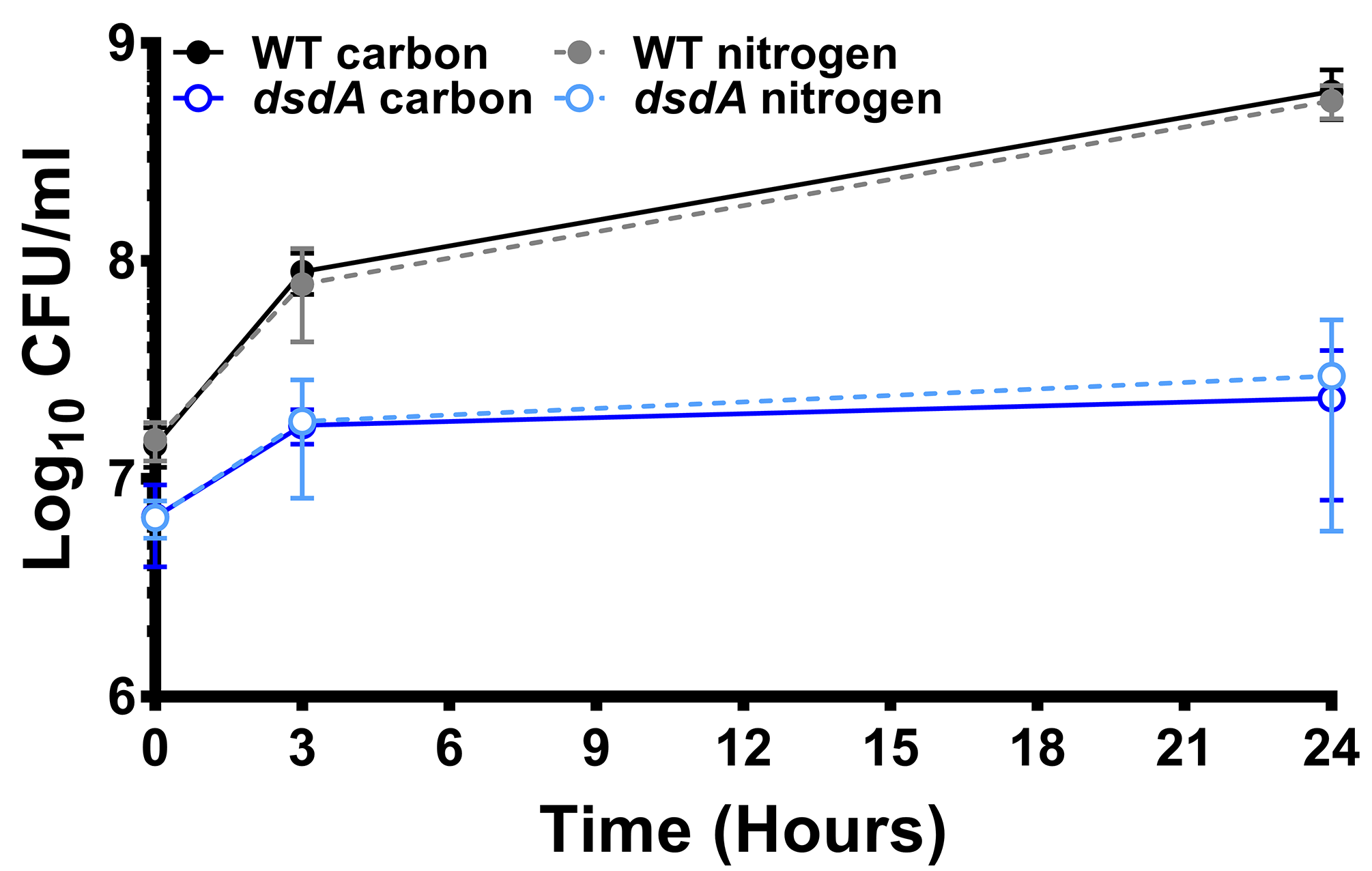

Supplement: FIG S1 [file mSphere.00020-19-sf001.tif]

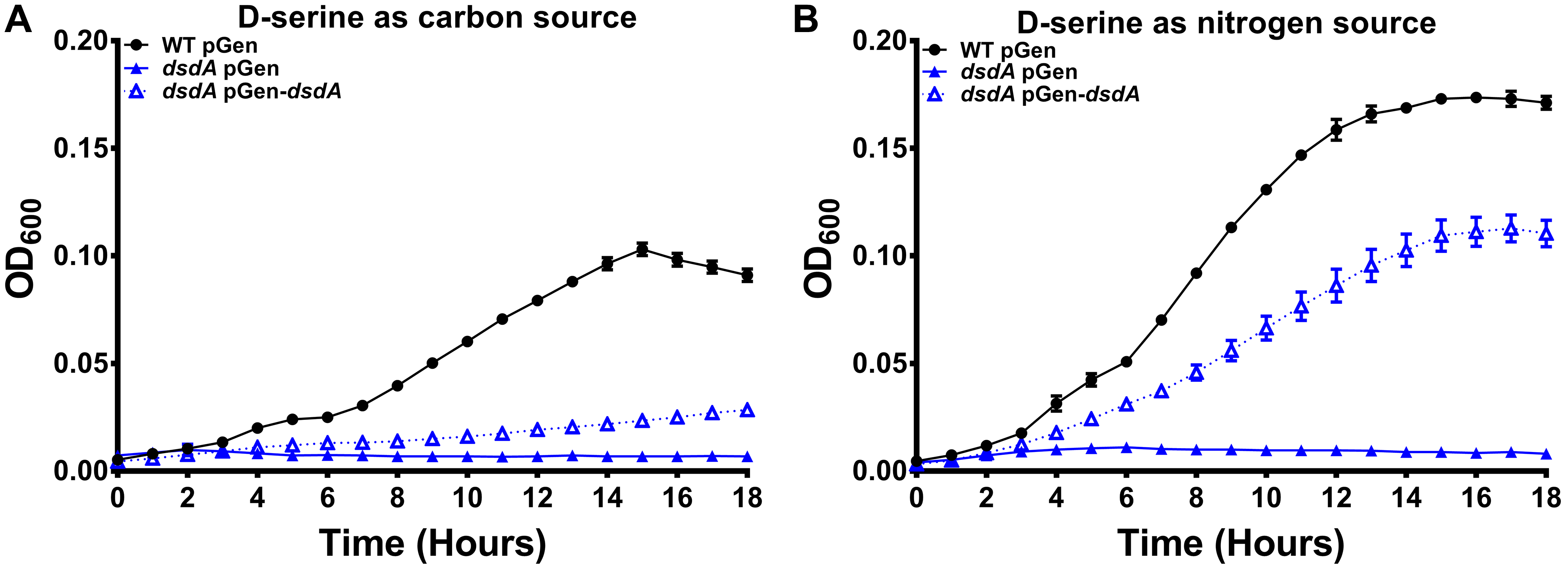

Supplement: FIG S2 [file mSphere.00020-19-sf002.tif]

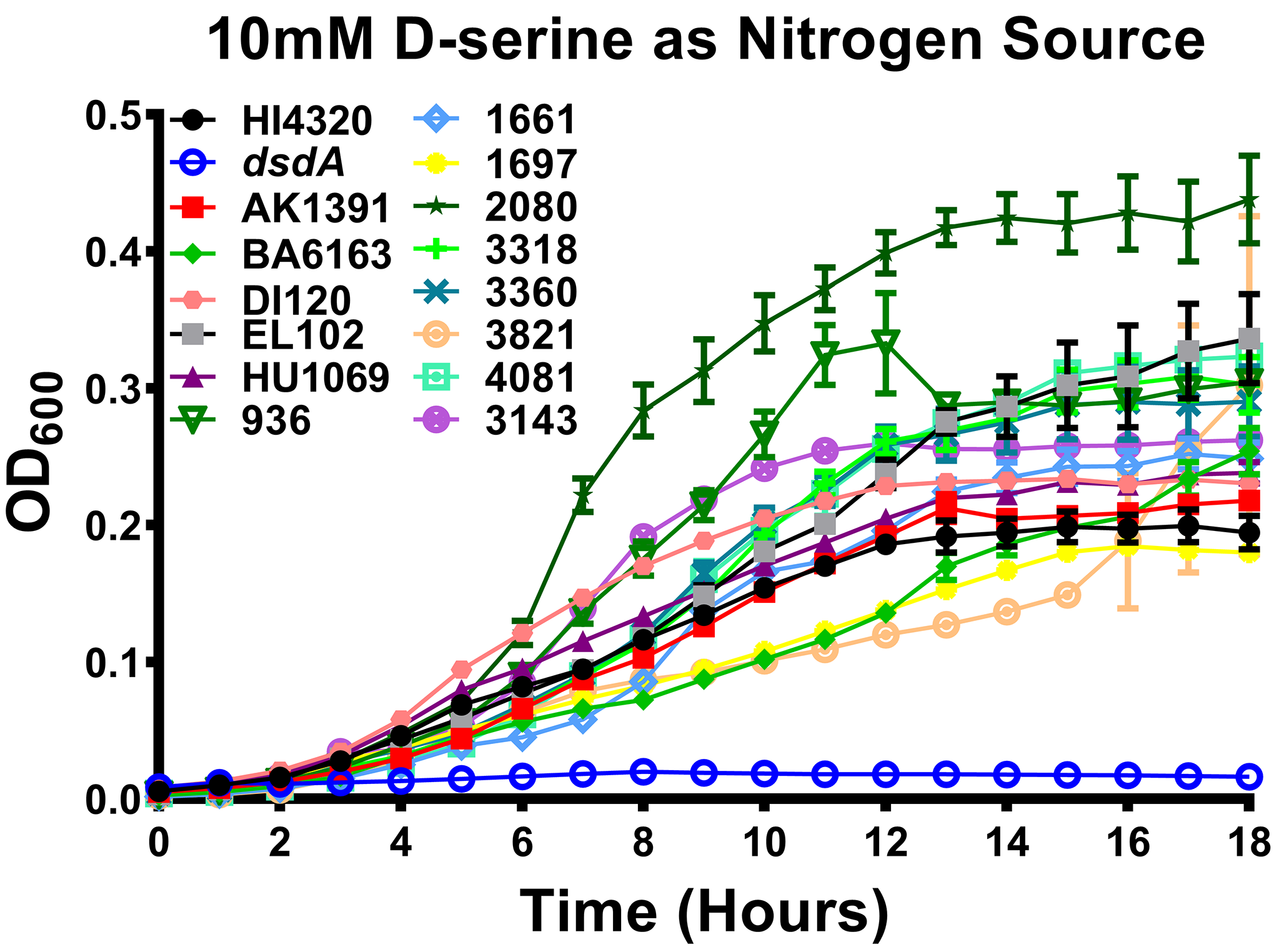

Supplement: FIG S3 [file mSphere.00020-19-sf003.tif]

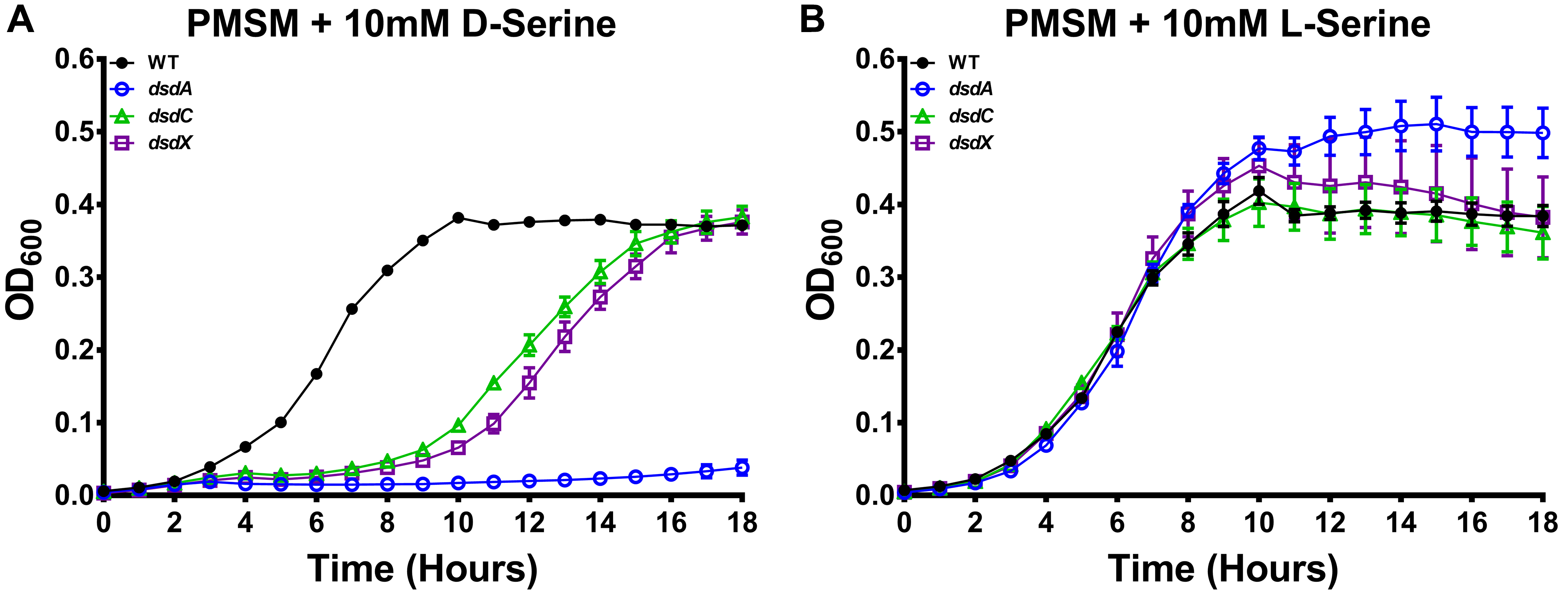

Supplement: FIG S4 [file mSphere.00020-19-sf004.tif]

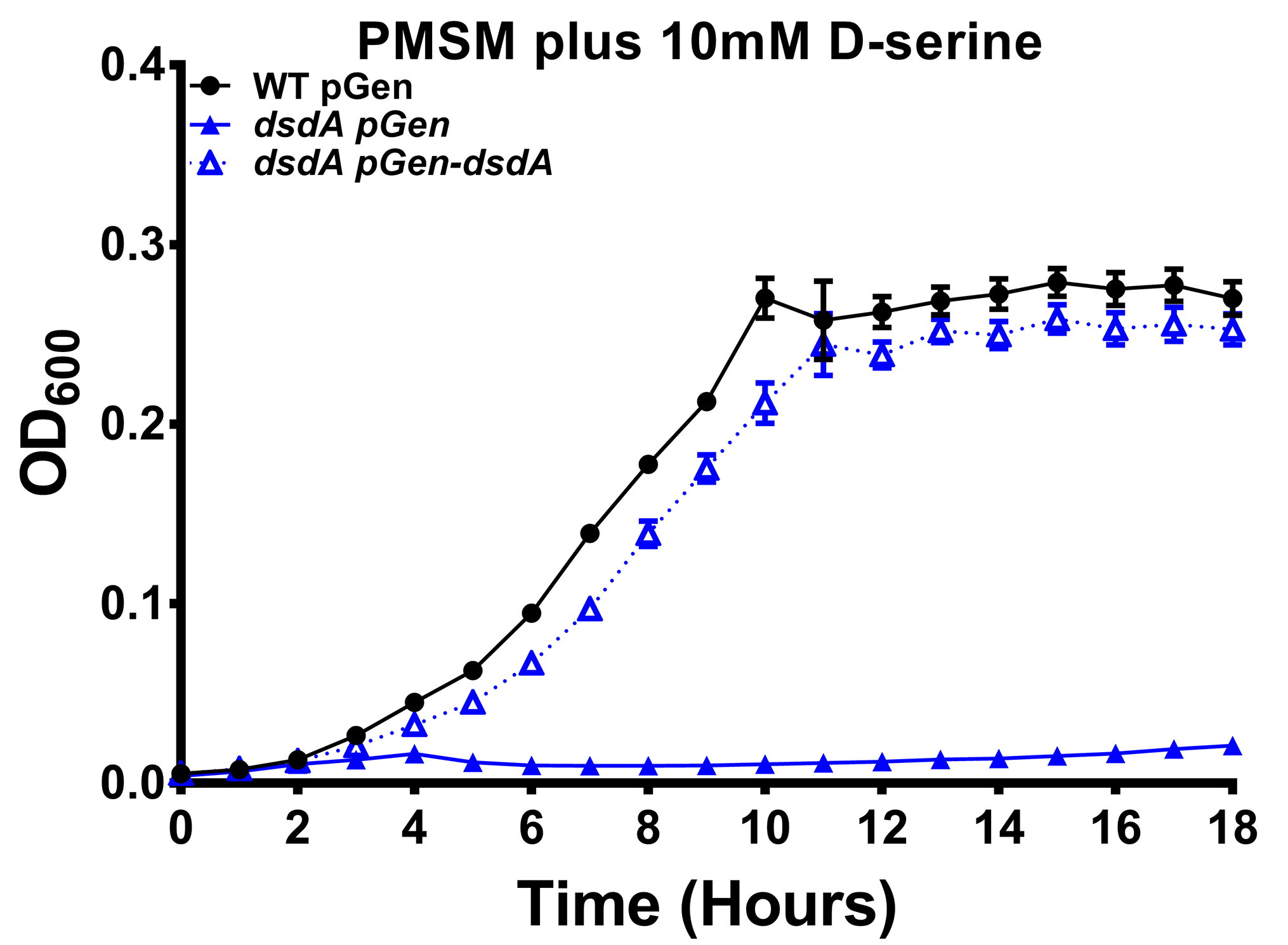

Supplement: FIG S5 [file mSphere.00020-19-sf005.tif]
